# Purchase Intention for Green Cars Among Chinese Millennials: Merging the Value–Attitude–Behavior Theory and Theory of Planned Behavior

**DOI:** 10.3389/fpsyg.2022.786292

**Published:** 2022-02-22

**Authors:** Lei Wang, Qi Zhang, Philip Pong Weng Wong

**Affiliations:** ^1^Department of Hospitality and Tourism, School of Management, Xuzhou University of Technology, Xuzhou, China; ^2^School of Management, Xuzhou University of Technology, Xuzhou, China; ^3^School of Hospitality, Sunway University, Bandar Sunway, Malaysia

**Keywords:** green car purchasing behavior, pro-environmental value, consumption value, value–attitude–behavior, theory of planned behavior, young generation

## Abstract

The value–attitude–behavior and the theory of planned behavior (TPB) appear to provide limited explanation for consumer green purchase behavior. This study aims to examine the relationship between pro-environmental value, consumption value, and TPB toward green car purchasing intention among the young Chinese generation. A total of 541 student responses were collected, and the results showed that altruistic value positively influenced subjective norm (SN) and perceived behavioral control (PBC), but negatively influenced green purchase attitude (GPA). Biospheric value positively influenced GPA and PBC. Function value and emotional value positively influenced GPA, respectively, and emotional value fully mediated the relationship between function value and GPA. Furthermore, GPA, SN, PBC positively influenced intention toward green car purchasing behavior, respectively, and SN mediated the relationship between GPA and intention. This study shows how pro-environmental value and consumption value can influence components of TPB in green car purchase intention.

## Introduction

Resource maintenance refers to the preservation or improvement of the resources that contribute to the enhancement of wellbeing; resources that are mainly physical, such as natural resources and manufactured products that underpin economic activities (Goodwin et al., 2014). However, over-utilization and consumption of natural resources by consumers is inevitable when there is accompanying rampant economic growth (Wang, 2020). In recent years, consumers perceived problems that are caused by environmental issues, for example, water pollution, haze, global warming, etc. have a severe negative impact on their living conditions (Wang et al., 2020c). This has resulted in increased awareness in the importance of the selection of eco-friendly products or services in their purchasing decision-making processes (Teeroovengadum, 2019).

The transportation industry accounts for about 60% of the world’s oil consumption and 25% of total world carbon dioxide (CO_2_) emissions (Silitonga et al., 2012). Specifically, the road transportation segment contributed to about 80% of the total consumption, which accounts for approximately 10% of greenhouse gas emission for the whole transportation sector (Silitonga et al., 2012). In China, the transportation industry accounts for 49.9% of oil consumption and 8.4% of CO_2_ emission (Sajjad et al., 2020). Therefore, green cars’ (e.g., electric vehicles and hybrid vehicles) policies were introduced and implemented in many countries due to the potential benefits of transforming the existing transportation industry toward a greener and cleaner future (Lim et al., 2019; Sajjad et al., 2020).

However, there has been a widely acknowledged gap between the attitudes and behaviors of consumers with respect to eco-friendly consumption (Kumar and Sreen, 2020). Despite consumers’ claims of their concerns for environmental issues and displaying positive attitudes, the claims have not been translated into green purchase behavior (GPB; Wang, 2020). The sales of green cars are still far behind traditional engine-powered cars (Lim et al., 2019). Hybrid cars only occupied 2.2% of the total new vehicle market in US market (Hur et al., 2013), and green cars make up only around 2% of vehicles sold annually in Malaysia (Lim et al., 2019). Even in the largest vehicle market, China, green cars only accounted for 4.82% of total vehicles sold in 2019 (Forward Business Information, 2020). This shows the lack of a deeper understanding on consumers’ green car purchasing behavior, due to the lack of a standardized definition and a solid foundation in research on GPB (Wang and Wong, 2021).

Prior studies applied the theory of planned behavior (TPB) to understand how antecedents influence intention in the context of green marketing (Wang and Wong, 2021). Certain studies adopted TPB as the underpinning theory and other studies included some of the components of TPB as a part of the research constructs (Elhoushy and Jang, 2020; Wang et al., 2020b). However, most of these studies often gave inconclusive or even controversial outcomes (Wang et al., 2019, 2020c). As TPB is a behavioral theory based on a causal process, it ignores other essential factors (Ulker-Demirel and Ciftci, 2020), such as impulse factors, feelings, private standards (Sniehotta et al., 2014); unconscious motives and spontaneous choices (Yuzhanin and Fisher, 2016); and personal decision criteria (Ulker-Demirel and Ciftci, 2020). This leads to the identification of the attitude–intention/behavior gap by some researchers who argue that factors influencing the magnitude of this gap have not been systematically investigated (Jacobs et al., 2018). Specifically, Wang et al. (2021b) indicated that the possible mediation effect of attitude between SN and green purchase intention (GPI) cannot be ignored in green marketing. However, how SN is linked to consumers’ attitude toward green cars purchasing behavior has been underexplored.

Apart from the impact of extending psychological predictors on GPB, values play a significant role in consumer’s pro-environmental decision-making (Tamar et al., 2020). Values are considered a trans-situational goal which varies in degree of importance and serve as a guiding principle in one’s life (Tamar et al., 2020), and are also relatively stable in the course of time (Jacobs et al., 2018). It has been recognized as an important driver of consumers’ product evaluations and future purchase decisions (Hur et al., 2013), and is considered as one of the critical antecedents for GPB (Tamar et al., 2020). However, the value–attitude–behavior models seem to be unable to explain behavior comprehensively (Jacobs et al., 2018).

Indeed, consumers’ pro-environmental value (i.e., altruistic value, biospheric value, and egoistic value) has frequently applied to measure one’s value toward GPB in some value-related theories (e.g., value–attitude–behavior model and value-belief-norm theory; Rahman and Reynolds, 2016). But compared to conventional studies, there is a lack of understanding how altruistic value influence on consumers’ GPB (Wang et al., 2020a). Meanwhile, previous studies have not distinguished biospheric value from altruistic value orientation, thus, leading to confusing results (Wang et al., 2020a). Indeed, past studies demonstrated the negative relationship between egoism and GPB might be less appropriate in eastern societies with high collectivistic value, such as China, Japan, and Korea (Wang et al., 2021b). Also, certain studies indicated that consumers’ consumption value (i.e., functional value, emotional value, and social value) significantly influenced their decision-making processes (Hur et al., 2013; Rasoolimanesh et al., 2020). However, few studies attempt to pursue a deeper understanding of the inter-relationship among sub-dimensions of consumption value which influence consumer purchase behavior (Rasoolimanesh et al., 2020), such as green cars purchasing behavior. Therefore, it is necessary to understand how various values influence the younger Chinese generation’s GPB toward green cars purchasing behavior.

The current study had assessed the influence of consumers’ values on the attitude component of TPB, specifically on how a merged value–attitude–behavior hierarchy (VAB) and TPB model influence consumers’ GPI of green cars in China. Most of the existing conceptual and empirical studies, and literature on TPB were focused on Western societies (Wang and Wong, 2021), and a small handful of studies discussed values in the context of green products in relation to green purchase attitude (GPA) and GPB (Jan et al., 2019). As yet, it is still unclear whether previous studies’ findings relating to values can be applied to more collectivistic countries (Wang et al., 2021d). Therefore, this study seeks to extend the existing knowledge of the influence of pro-environmental values and consumption values on GPB and the influence of GPA, subjective norm (SN), perceived behavioral control (PBC) and GPI on the purchasing behavior of green cars among Chinese millennials.

## Conceptual Model and Hypotheses Development

### The Value–Attitude–Behavior Model

Value–attitude–behavior (VAB) model is a classical model in the literature of social psychology, which investigates the relationship between value and behavior through attitude (Jan et al., 2019). Specifically, the VAB model summarizes specific extant literature on natural food consumption and leads to the development of corresponding hypotheses (Jacobs et al., 2018). Since then, researchers have employed the VAB model in different contexts, such as local food consumption (Zhang et al., 2020), green customer loyalty (Hur et al., 2013), green hotel patronage (Rahman and Reynolds, 2019), and pro-environmental behavior (Tamar et al., 2020).

### The Influence of Pro-environmental Values on GPB

An individual’s preference of GPB is affected by environmental values (Bautista et al., 2020) which play a vital role in influencing pro-environmental behavior (Rahman and Reynolds, 2016) and it is used as a predictor of consumer attitude and behavior toward green products (Bautista et al., 2020). Environmental values represent an individual’s principles on the importance of pro-environmentalism and sustainability (Barboza and Filho, 2019) and it can explain the individual’s motivation to engage in green campaigns (Bautista et al., 2020). This corresponds with the TPB model which posits that environmental beliefs shape attitude, which is then translated into GPI (Rahman and Reynolds, 2016).

#### Altruistic Value

The classical value-belief-norm theory which supports the moral norm activation theory of altruism (Schwartz, 1977) argued that an individual who exhibits altruistic behavior is able to help others because he/she responds to the activation of his/her personal moral norms, when particular conditions pose threats to others; and believes his/her actions might lead to the avoidance of negative consequences (Wang et al., 2020a). This theory has been applied by (Stern, 2000) to support his proposed value-belief-norm theory of environmentalism (VBN) in green marketing. Based on Stern (2000)‘s theory, individuals who act altruistically are able to relate to pro-environmentalism due to their beliefs that when certain conditions pose threats to others’ living situations, the actions they could perform might help others to avoid negative outcomes (Wang et al., 2020a).

Accordingly, altruistic value refers to a desire to benefit others instead of looking after oneself’s interest (Bautista et al., 2020). An individual’s feeling of what is ethically right is composed of his/her commitment to creating the best outcome for others (Teng et al., 2015), irrespective of what others individuals think (Wang et al., 2020a). Meanwhile, altruistic value includes the demonstration of accomplishing something good for others without expecting anything in return (Rahman and Reynolds, 2019). Altruistic value has been found to be stronger among individuals who partake in certain GPBs for the welfare of others considering their own interests (Rahman and Reynolds, 2016; Bautista et al., 2020; Li et al., 2020). As such, altruistic value can be considered an antecedent for GPB (Kaufmann et al., 2012).

Certain studies have highlighted a significant causative path from altruistic value to components of TPB on GPB. For example, Birch et al. (2018) found that altruistic value positively and directly affected one’s attitude of opting for green products. Likewise, Wang et al. (2020a) extended the value-belief-norm theory model to predict consumer GPB and indicated that altruistic value is the most important value dimension significantly influenced traveler’s GPA. Meanwhile, Eid et al. (2020) revealed the significant positive effect of altruistic value on consumers’ norms toward green hotels visitation, while Obrenovic et al. (2020) investigated an individual’s knowledge-sharing behavior and demonstrated the high explanatory power of altruistic value as a determinant of SN in Croatia. Additionally, Rahman and Reynolds (2016) found that altruism value positively influenced consumers’ PBC in terms of willing to sacrifice for green products and willingness to pay a premium. Likewise, Teng et al. (2015) adopted the TPB model and found that altruistic value positively affected PBC in the selection of green hotels in Taiwan, China. However, studies on altruistic value are scarce when compared to conventional studies (Wang et al., 2020a). Hence, this study postulates the following hypotheses:

*H1*: Altruistic value positively influences GPA.

*H2*: Altruistic value positively influences SN.

*H3*: Altruistic value positively influences PBC.

#### Biospheric Value

The main drawback of moral norm activation theory of altruism is it focus mainly on GPB in the private sector (Stern, 2000), for example on consumers’ personal GPB related to household and disposable products practices that have a negative environmental impact (Wang et al., 2020a). This theory ignores non-activist GPB in extant literature (Stern, 2000), as most of previous studies have not differentiated biospheric value from altruism orientation (Rahman and Reynolds, 2016). According to Stern (2000), GPB can reasonably be characterized by concerns on the effect of materials or energy production on the ecosystems or the biosphere itself. Hence, the biospheric value provides a distinct support for preserving the environment (Wang et al., 2020a), which emphasizes the welfare of the environment only (Rahman and Reynolds, 2016). Therefore, individuals who possess biospheric values exhibit more concern for animals, plants, and other natural resources, and can be regarded as pro-environmentalists (Hughner et al., 2007; Wang et al., 2020a).

Biospheric value should be considered as the most important principle leading to GPB (Wang et al., 2020a), and it should provide better explanatory power when compared to altruistic value in GPB predictions (Rahman and Reynolds, 2016). However, the concept of biospheric value in green literature is still new and remains unresolved empirically (Wang et al., 2020a). Certain studies on GPBs have demonstrated how biospheric value positively influences attitude, SN, and PBC. Rahman and Reynolds (2019) indicated that biospheric value represents an inherent concern for the nature and environment, thus, biospheric value positively influenced consumers’ GPA toward green products. Meanwhile, Wang et al. (2020a) reported a positive relationship between biospheric value and GPA among 248 Chinese tourists. Furthermore, Bamberg (2003) found that consumers who are more concerned about environment more likely to display higher SN toward GPB, while Paul et al. (2016) reported similar results that consumers who are concerned about environment are more likely to be influenced in their SN toward GPB in India. In addition, Rahman and Reynolds (2016)‘s findings that biospheric value positively influenced consumers’ PBC in visiting green hotels, and Rahman and Reynolds (2019) revealed a positive direct effect of biospheric value on consumers’ PBC in terms of willingness to pay more for green products. To lend further support to the importance of biospheric value in GPB literature, the following hypotheses are proposed:

*H4*: Biospheric value positively influences GPA.

*H5*: Biospheric value positively influences SN.

*H6*: Biospheric value positively influences PBC.

#### Collectivistic Value

Egoism is another value that can significantly influences one’s GPB (Rahman and Reynolds, 2016). This value focus on maximizing individual outcomes based on self-interests (Wang et al., 2020a), and involves values, such as obedience, self-discipline, and family security, and they can negatively influenced pro-environmental norms and behaviors (Stern, 2000). Egoistic values may provide an important basis for principled opposition by some individuals to environmental movement objectives, but the ways egoistic values affect behavior are not well understood (Wang et al., 2020a). Past studies affirmed egoistic value is connected to environmental beliefs and behaviors (Stern, 2000; Rahman and Reynolds, 2019), as GPB generally entails a clash between short-term personal benefits and long-term collective concerns (Rahman and Reynolds, 2019). Therefore, collectivism and individualism are at two opposing ends of this value spectrum in green marketing (Kumar and Sreen, 2020; Wang et al., 2020a).

Accordingly, collectivistic value refers to a collective need to protect the environment in order for all society to prosper (Chen, 2013), as well as emphasizing interdependence, group-orientation goals, cooperation, and minimal competition (Wang et al., 2020a). In contrast, individualistic value refers to the moral stance, political philosophy, ideology, or social outlook that stresses “the moral worth of the individual” (Gagnier, 2010). It is characterized by independence, self-reliance, freedom of choice, and a high level of competition (Wang et al., 2020a). Thus, individuals who have a strong, selfish and competitive orientation are less likely to perform pro-environmental behavior, and individuals who have satisfied their own needs are more likely to perform GPBs and are more focused on pro-environmental issues (Wang et al., 2020a).

Previous studies conducted in individualistic countries, demonstrated that egoistic value negatively influences consumers perceptions to reduce car use in Czech Republic (De Groot and Steg, 2007), while Bouman and Steg (2019) reported similar results in the Netherlands. It is yet unclear whether those findings also apply to more collectivistic countries (Wang et al., 2021d). Specifically, Wang et al. (2020a) argued that applying egoistic/individualistic value for consumers who reside in certain Eastern nations (e.g., China, Korea, and Japan) is not appropriate, due to the highly collectivistic values practiced in these societies when compared to most Western countries. Thus, a single measurement of egoistic or individualistic value may not suit all settings, but applying a reliable measurement of collectivistic value seems to overcome such problems (Wang et al., 2020a).

Certain studies revealed how collectivistic value significantly influences consumers’ GPA, SN, PBC and GPB. For example, Wang et al. (2020a) applied the value-belief-norm theory to consumers’ green visit intention in China using an online sampling indicated a positive direct effect of collectivistic value on consumers’ attitude. Wang et al. (2021b) further explored the relationship between value and consumers’ GPI showed that collectivistic value had a significantly positive relationship with GPA. Kumar and Sreen (2020) explored the relationship between internal/external value and GPB found that collectivistic value positively correlated with Indians consumers’ attitude, SN and PBC. Tsen et al. (2006) investigated the relationship between consumers’ collectivistic value and control beliefs in Malaysia and demonstrated that collectivistic value positively influenced one’s willingness to pay for green products. Based on above considerations, the following hypotheses were proposed for testing:

*H7*: Collectivistic value positively influences GPA.

*H8*: Collectivistic value positively influences SN.

*H9*: Collectivistic value positively influences PBC.

### The Influence of Consumption Values on GPB

Consumer value is considered a crucial factor for determining a product or service’s attractiveness (Hur et al., 2013). As consumer value is inherent to the experience in the use of a product or a service, a consumer’s perceived value cannot be determined objectively by the providers (Hur et al., 2013). A number of marketing related studies have examined and confirmed the significant effect of consumer value on different aspects of one’s purchasing behavior (Zaidi et al., 2019; Rasoolimanesh et al., 2020).

The theory of consumption values demonstrated that functional, social, emotional, epistemic and conditional values as key dimensions of an individual’s perceived values that affect one’s purchasing behavior (Sheth et al., 1991). However, researchers have generally omitted epistemic and conditional values as being too transient (Rasoolimanesh et al., 2020), and it is not always practical for researchers to include all five values when the choice situation might be driven by a smaller set (Sheth et al., 1991). Most previous studies take conditional value into account with other value dimensions; however, conditional value is not a value itself, but it reflects the effect of a product’s utility in the particular situations and circumstance (Caber et al., 2020). Meanwhile, epistemic value can be incorporated into emotional value, since this value is related to curiosity, novelty, and cognition obtained from the products or service (Caber et al., 2020; Rasoolimanesh et al., 2020). Overall, a parsimonious explanation of consumption value indicates that consumers assess a product or service, not just in functional terms of expected performance, but also in terms of the enjoyment of pleasure derived from emotional value and the social consequences of what it communicates about other consumers (Sweeney and Soutar, 2001). This multidimensions scale has been found to be reliable and valid in a variety of purchase situations (Hur et al., 2013), which provides a suitable framework to explore the effect of consumption value on green cars purchase intentions.

#### Functional Value

Functional value refers to the rational and economic evaluations made by consumers (Carlson et al., 2019), because it is associated with the practical or technical benefits consumers can obtain by using a product or service (Hur et al., 2013). Individuals perceived functional value or economic utility of a product or service which is derived from the product attributes, such as durability, reliability, price (Jan et al., 2019), and quality (Zaidi et al., 2019). Thus, functional value is associated with the perceived benefits of a product or service’s functional, utilitarian, and physical performance (Caber et al., 2020) and was thought to be generated by a product or service’s salient attributes (Zaidi et al., 2019). Overall, the functional value of the product or service refers to the net utility that is derived from the perceived quality attributes and more importantly, the price of the product (Jan et al., 2019).

According to Hur et al. (2013), the fuel-efficiency of green cars can be very attractive to some consumers, because similar to the economic value, the functional value of green cars refers to perceived economic utility of purchasing that is derived from the attributes (e.g., reduce energy usage and saving natural resources) of green cars (Jan et al., 2019). Bjerkan et al. (2016) explored the incentives influence promoting battery electric vehicle choice in Norway and indicated that functional value attributes, for example, value-added tax exemption, purchase tax exemption, free vehicle license, and free parking, significantly influenced potential consumers’ attitude, perceptions, and purchase behavior. Similarly, Sierzchula et al. (2014) explored the consumers’ attitude–action gap related to green cars in US and demonstrated that functional value significantly influences consumers’ purchase attitude and behavior. Thus, the following hypothesis was proposed:

*H10*: Functional value positively influences GPA.

#### Emotional Value

The emotional value refers to the utility derived from affective feelings or states that a product or service generates (Rasoolimanesh et al., 2020). It aims to meet an individual’s mental or psychological needs of a product or service (Hur et al., 2013). The buying process of product or service itself will bring about positive or negative affective feelings (Caber et al., 2020). Emotional value can be considered as the most important predictor of behavioral intention in literature because although an individual may not seek emotional benefits intentionally during the consumption experience, positive/negative feelings aroused unintentionally from the experience play an important role in further decision-making at a subconscious level (Hur et al., 2013). According to Rasoolimanesh et al. (2016), emotional value can be categorized under hedonic orientation and novelty. For instance, consumers with increased environmental concerns may feel optimistic about using green cars rather than conventional cars because they feel they are doing the right thing to solve environmental issues (Hur et al., 2013). Meanwhile, consumers could also receive positive feelings because of the perception they are adopting some novel innovations when they are driving green cars compared to conventional cars.

Hur et al. (2013)’s study results indicated that both dimensions of emotional value positively influenced consumers to buy hybrid cars, and Rasoolimanesh et al. (2020) found similar results among hotels’ consumers to visit guesthouses. Moons and De Pelsmacker (2012) investigated antecedents influence electric car usage intention in Belgium using a snowball sampling with a sample of 1,202 respondents. It resulted in a significant relationship between emotional value and purchase of innovative products behavior as emotional value can be perceived as an important cognitive consideration in the usage intention formation process. A recent study by Joshi et al. (2021), who applied the TPB to predict consumers’ GPI that involved a sample of 387 respondents, showed that emotional value had a significant positive relationship with GPA, and consequently, GPI. Thus, the following hypothesis is proposed:

*H11*: Emotional value positively influences GPA.

Although the functional and emotional values are conceptually related, however, previous studies’ models tend to ignore the correlation between these two values (Rasoolimanesh et al., 2020). Theoretically, the cognitive appraisal theory of emotions stresses that the evaluation of the outcomes of product or service usage causes an emotional or affective response (Ladhari et al., 2017). According to Lee et al. (2010), the emotional value can promote one’s satisfaction and intention as of satisfying his/her needs in terms of emotions. In other words, the emotional value is connected to functional attributes of the product or service and emotional consequences are raised from adopting a product or service (Hur et al., 2013). Thus, achieving good quality functional services are expected by individuals, providing high-quality product or services and meeting their expectations result in positive feelings (Rasoolimanesh et al., 2020). An individual’s emotions are evoked by his/her rational and economic evaluation of the product or service, and the greater functional value triggers one’s emotional value perception, subsequently, enhances the level of his/her perception and intention (Rasoolimanesh et al., 2020).

Certain studies demonstrated how emotions are evoked by the consumers’ rational and economic evaluation of the product or services; for example, Ladhari et al. (2017) indicated that perceived service performance positively influenced emotions, which then influence consumers’ perception and intention on products. Babin et al. (2004) explored the cognitive and affective determinants of retail patronage and demonstrated that functional value attributes (i.e., utilitarian values) positively effect on hedonic shopping values and service quality is positively correlated with emotional value (Amin et al., 2013). Thus, considering the above findings, the following hypothesis is proposed:

*H12*: Emotional value mediates the relationship between functional value and GPA.

#### Social Value

Social value is derived from the ability of the product or service to reinforce or improve the consumer’s social self-concept (Rasoolimanesh et al., 2020). According to Sheth et al. (1991), social value refers to “perceived utility acquired from an association with one or more specific social group, that is, it was measured through the product’s association with various reference groups of customers.” Thus, social value can be obtained when consumers feel they are connected to others by using certain products or services (Hur et al., 2013). Social value is considered to be connected to self-image (Bautista et al., 2020), since interactions between consumers/staffs/consumer/employees can have a profound effect on ones’ purchasing experiences (Rasoolimanesh et al., 2020).

Hur et al. (2013) argued that consumers may feel connected and belonging to an environmentally conscious group *via* purchasing hybrid cars, and thus, benefit others. Another study by Zaidi et al. (2019) found that social value positively influence consumers’ perceptions toward GPI. In addition, Caniëls et al. (2021) extended the theory of consumption value to youths GPB in Poland which showed that social value had a significantly positive relationship with GPA. Hence, the following hypothesis was developed:

*H13*: Social value positively influences GPA.

Although certain marketing literature have successfully highlighted the relationship between social value and emotional value (Nkaabu et al., 2017; Rasoolimanesh et al., 2020), the tendency in green marketing studies has been to regard these concepts as independent of each other. In contrast with emotional value which always plays a significant role in determining consumers’ attitude and behavior (Eid, 2015), social value seems to have a weak effect on consumers’ feelings and behavior (Kim et al., 2019). Rasoolimanesh et al. (2020) concluded that researchers should consider emotional value be treated as mediator between social value and one’s attitude/behavior, because socialization and communication may enhance consumers’ self-esteem and social status as reasons for the positive influence of social value on emotional value (Kazakevičiūtė and Banyte, 2012).

Few studies performed in-depth analyses on the effect of social value on emotional value in consumer GPB, but certain studies demonstrated that emotional value mediates the relationship between social value and consumers’ attitude or behavior. For example, Nkaabu et al. (2017) indicated that social value has a positive effect on consumers’ hedonic value toward purchase intention. Rasoolimanesh et al. (2020) demonstrated that social value positively influenced consumers’ evaluation of emotional value generated on traditional guesthouses satisfaction. In addition, Wu et al. (2018) found that social value positively affected intention of social online shopping. Hence, considering the above, the following hypothesis was proposed:

*H14*: Emotional value mediates the relationship between social value and GPA.

### The Theory of Planned Behavior

Similarly, TPB is another popular theory in the literature of the consumer decision-making process (Wang et al., 2021c), which was extended from theory of reasoned action (TRA). The main difference between TRA and TPB is that TPB consider one’s behavior cannot to be purely based on volitional factors (Wang et al., 2020b). Thus, non-volitional factors, such as those identified in the perceived behavioral control (PBC) variable of TPB, were included as an added predictor that extended TRA boundaries (Wang, 2020). The TPB model comprises of four constructs, namely, attitude, subjective norm (SN), PBC and intention, and eventually the behavior (Wang et al., 2021a). Many researchers applied TPB to estimate consumer’s green/purchase behavior, such as green hotel selection (Wang et al., 2019), destination choice (Wang et al., 2021c), and eco-label food consumption (Ateş, 2021).

Attitude or GPA is the most important predictor in TPB that influence consumers’ GPBs due to its stability and consistency (Wang et al., 2019). GPA refers to an individual’s positive/negative and favorable/unfavorable evaluation of a given behavior (Wang and Wong, 2021). GPA incorporates the judgment on whether the given behavior under consideration is good, bad, or indifferent, regardless of whether or not the consumer wants to perform the behavior that they are environmentally concerned about (Wang and Wong, 2021). Individuals may recognize the seriousness of environmental issues are actually caused by excessive use of natural resources and thus, their environmental awareness can instill positive attitudes toward GPB (Wang and Zhang, 2021). Many studies on green marketing have shown how GPA positively influences GPI (Wang et al., 2020a,c). Therefore, the following hypothesis was proposed:

*H15*: GPA positively influences GPI.

Subjective norm refers to the cognizant social pressure to perform or not to perform a specific behavior (Ajzen, 1991). In other words, the subjective norm is the perceived opinions of the significant others who are close to an individual and who influence his/her decision-making process (e.g., relatives, close friends, business partners, or co-workers/colleagues; Wang and Wong, 2021). Fundamentally, SN is the feeling or moral obligation of consumers, and it is a powerful motivator of environmental caring behavior (Wang et al., 2019). The social dynamic in which individuals associate with other individuals is by sharing the same values, thoughts, and beliefs (Sinnappan and Rahman, 2011). Thus, individuals are generally concerned about whether significant others would approve or disapprove of the given behavior (Wang et al., 2019). Certain studies have highlighted a significant causative path from SN to intention (Paul et al., 2016; Liu et al., 2020). Hence, the following hypothesis was proposed:

*H16*: SN positively influences GPI.

Nevertheless, Han and Stoel (2017) demonstrated that the SN seems to be the weakest component of TPB in previous studies. For instance, SN has been employed in some previous studies as a predictor for consumer GPB, resulting in an insignificant correlation with GPI (Sutikno and Indarini, 2020; Wang and Wong, 2021). In addition, previous studies showed that there is a complicated relationship between SN, GPA, and intention. Certain studies revealed that there is a positive relationship between SN, GPA, and GPI in green marketing; for example, Wang et al. (2019) indicated that there is an insignificant relationship between SN and GPI, however, SN had a significant influence on GPA, subsequently, GPI. While Wang and Wong (2021) demonstrated that SN had no role in determining GPI, but SN had a significant influence on GPI *via* GPA. In other words, GPA plays a mediating role in the relationship between the SN and GPI. Thus, the following hypothesis was developed for testing:

*H17*: GPA mediates the relationship between SN and GPI.

**Figure 1:**
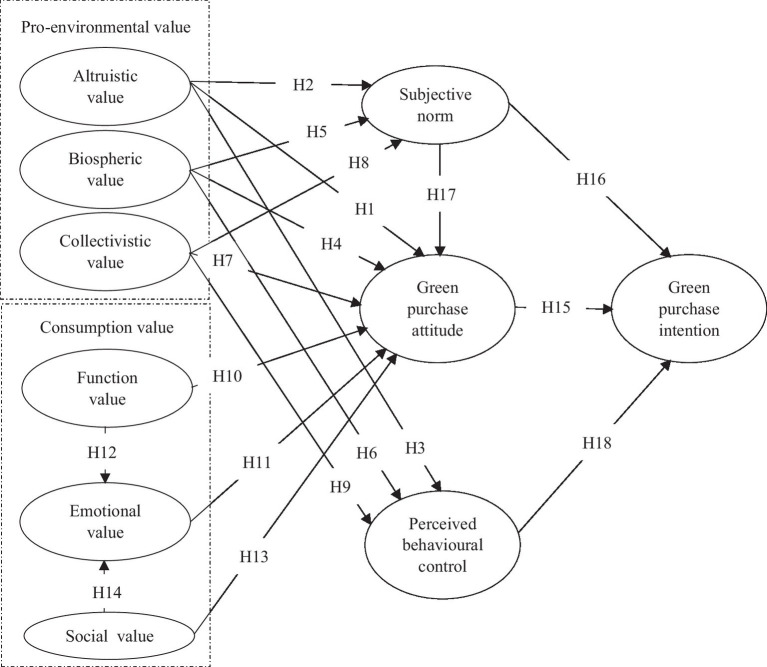
Theoretical research model.

Perceived behavioral control refers to the perception of how difficult or challenging it is to perform a certain behavior (Wang et al., 2019). It depends on both motive and ability aspects, which incorporates previous experiences and anticipated hindrances (Paul et al., 2016). It also involves the perception of how well individuals can control non-rationale factors that may encourage or oblige specific activities (Wang and Wong, 2021). PBC should be considered as an important predictor in TPB model, due to its high explanatory capacity in a situation with perceived constrains compared to normative orientation theories (e.g., norm activation model and value-belief-norm theory of environmentalism; Steg and Vlek, 2009). Certain studies on GPB have demonstrated how PBC positively influences GPI/GPB (Teeroovengadum, 2019; Wang and Wong, 2021). However, other researchers have utilized PBC to predict consumer GPB, which resulted in ineffective PBC for consumer GPI (Eid et al., 2020; Sutikno and Indarini, 2020). Thus, the following hypothesis was proposed for testing, and the theoretical research model (Figure 1) for this study was established based on above mentioned literature.

*H18*: PBC positively influences GPI.

## Materials and Methods

### Data Collection

In social science, researchers generally cannot easily acquire an accurate sampling frame from companies or locate appropriate respondents to answer the research questions (Saunders et al., 2011). Thus, the non-probability sampling is often used as an alternative technique to select samples based on subjective researcher judgment (Sekaran, 2006). A convenience sampling method was utilized to collect samples in this study due to the well-known advantages, such as easy accessibility, availability at a given time, geographical proximity, and higher willingness to participate (Dörnyei, 2007). More specifically, this method allows researchers to have easier access with potential research subjects of the population (Etikan et al., 2015).

The target respondents were the younger generation in China, specifically young Chinese generations that showed robust market purchasing power. According to Bahl and Kumar (2019), the younger generations play a significant role in determining forthcoming market segmentation as they will shape a different consumption pattern in the future. Wang et al. (2021b) indicated that 42% of Chinese young generation (i.e., aged between 18 and 25) expressed high intention to purchase novel products and services in the future, which is higher than the Americans, Europeans and Japanese. This phenomenon also exists in new energy vehicles industry. Based on J. D. Power (2021), generation Z has the highest purchase intention among all age groups. Consumer purchase intention of new energy vehicles increase to an all-time high of 16% in 2018, specifically, generation Z has the highest intention (J. D. Power, 2021). Moreover, young generations are better educated and are more concerned and knowledgeable about environmental issues (Varah et al., 2020), and they like sustainable lifestyles and are often ready to adopt innovative and green technology and green products and services (Jaiswal and Kant, 2018). In addition, the household decision for purchasing a new car or the second is increasingly influenced by young and educated adults in their family (Jaiswal et al., 2021).

A convenience sampling method was used to collect data at six undergraduate universities in Xuzhou, Jiangsu province, China. The total number of undergraduate students in Jiangsu is more than 1.1 million which ranks the third highest in China, and Xuzhou occupied almost one-fifth of the total (Wang et al., 2021c). A network of contacts at universities throughout Xuzhou cooperated in distributing and returning the questionnaires. All of the contacts were university lecturers, assistant professors, and associated professors. Each contact received a packet containing between 100 and 200 questionnaires, depending on the number of students with whom they interacted. Questionnaires were distributed between March and May to students through an online system who completed them in the classroom, and participation was voluntary and were not compensated for their participation. The students were from diverse departments (e.g., education, hospitality and tourism, marketing, global business, English language, literature, sports, and economics). A total of 541 usable questionnaires were collected which exceeded Hair et al. (2010) which states that sample size of more than 200 have been found to provide an acceptable margin of error. This also corresponds with Kline (2015) suggestion that a minimum sample size of 200 respondents and between 10 and 20 cases per parameter is required for structural equation modeling, as well as Cochran’s formula that determined a minimum of 384 sample size is recommended for target population which is unknown (Sarmah et al., 2013).

### Measures

The research instrument adopted was the self-administered questionnaire. The questionnaire was designed in four sections. The first section included pro-environmental values: altruistic, biospheric and collectivistic value. Six items belonging to altruistic value were adapted from Mas’od and Chin (2014); six items used to measure biospheric value were adapted from Teng et al. (2015) and Rahman and Reynolds (2016); six items belonging to collectivistic value were adapted from Wang et al. (2020a). The second section included the consumption values: function, emotional and social value. Ten items belonging to function value and five items used to measure emotional value were adapted from Hur et al. (2013) and Rasoolimanesh et al. (2020); six items related to social value were adapted from Hur et al. (2013), Caber et al. (2020), and Rasoolimanesh et al. (2020). Third section items were used to assess the TPB’s components: SN, GPA, PBC and GPI. Three items used to measure SN and three items belonging to PBC were adapted from Wang and Wong (2021), four items used to assess GPA and three items used to measure GPI were adapted from Wang et al. (2020a). Lastly, the fourth section elicited relevant demographic characteristics. All of measurement items were evaluated using a five-point Likert scale, ranging from “strongly disagree” to “strongly agree.” All questionnaire items were translated into Chinese using the back-translation method by three bilingual experts to ensure translation accuracy. A pretest was conducted involving 40 respondents to ensure the usability and validity of the developed instrument and to prevent any problems that may affect the quality of the collected data.

### Common Method Bias Issues

Lastly, the Common Method Bias (CMB) is considered as another major concern in survey studies (Hulland et al., 2018). In this study, all respondents from different majors completed the questionnaires to reduce CMB impact from homogeneous issues; second, the measures used multiple scale types, containing differential, bipolar, semantic, and Likert. Podsakoff et al. (2003) indicated that a common latent factor can be used to examine CMB. During CFA process, a latent variable was included in model by connecting it to all observable factors, and the standardized regression evaluated the new model before comparing it with the original model showed similar results after comparison. Finally, Harman’s single factor test was performed to determine the existence of CMB in influencing results. The results showed that single factor score obtained a variance of 40.526%, indicating CMB is not a pervasive issue for this study.

### Data Analysis and Results

The Statistic Package for Social Science (SPSS) provides a vast array for programs for univariate, bivariate and multivariate statistical analysis and it is considered the most widely available and used comprehensive statistic calculation software for marketing (Malhotra and Birks, 2007; Green and Salkind, 2010). Thus, the SPSS 19 version was utilized for the descriptive statistics for this study. The next step performed was a confirmatory factor analysis (CFA) and structural equation modeling (SEM) test with AMOS. According to Risher and Hair (2017), covariance-based application (e.g., CB-SEM or LISREL) is based on the common factor model, indicating the analysis is based on the common variance derived from the covariances between all variables in the structural model, determines how well the model can estimate the covariance matrix for the sample data with the ultimate goal of confirming theory (Hair et al., 2014). In contrast, variance-based application (e.g., PLS-SEM) uses a composite model, in which optimum solutions are based on the total variance of all indicators in the model (Risher and Hair, 2017). The main objective of variance-based application is to minimize unexplained variance in the dependent variables, thus, it is well suited for analyzing predictive, complex models with a large number of variables and relationships (Hair et al., 2019). Therefore, the CB-based CFA and SEM were adopted for this study as this study attempts to explore the effect of various dimensions of pro-environmental value and consumption value on youth green car purchase intention based on VAB model and TPB model.

#### Descriptive Statistics

SPSS version 19 was employed for the descriptive statistics and Table 1 displays the segmentation for the demographic characteristics of the samples. Of the 541 valid questionnaires returned, 83.2% were female, 50.5% respondents reported that they were junior candidates. 32.3% of the respondents are 20 years old, and most students’ monthly living expenses are between 1,501–2,000 yuan (36.8%).

**Table 1:** Sample characteristics (*N* = 541).

Items	Characteristic	Frequency	Percentage (%)
Age	Below 18	18	3.3
	18	20	3.7
	19	76	14.0
	20	174	32.3
	21	154	28.5
	22	56	10.4
	23	30	5.5
	Above 23	13	2.4
Gender	Female	450	83.2
	Male	91	16.8
Education level	Freshman	223	41.2
	Sophomore	5	0.9
	Junior	273	50.5
	Senior	22	4.1
	Master and above	18	3.3
Living expenses	Below 1,000	46	8.5
	1,000–1,500	189	34.9
	1,501–2000	199	36.8
	2001–2,500	69	12.8
	2,501–3,000	12	2.2
	Above 3,001	26	4.8

#### Confirmatory Factor Analysis

In the measurement model, researchers should accept items with loadings more than 0.5 (Hair et al., 2010). Thus, factor loadings below 0.5 were dropped (i.e., BV5, BV6, CV6, SV5, and SV6) before finalizing the validity and reliability of the remaining items. Besides, to test convergent validity of the model, the composite reliability (CR) for each variable were higher than the thresholds of 0.7, and the average variance extracted (AVE) were higher than the minimum criteria of 0.5 as suggested by Hair et al. (2010) (See Table 2). Moreover, to assess discriminate validity, heterotrait–monotrait ratio of correlations (HTMT) was considered. As shown in Table 3, the threshold value for HTMT should less than 0.9 or even less than 0.85 (Henseler et al., 2015), thus indicating that discriminant validity exist according to HTMT test.

**Table 2:** Construct validity.

Variables (Cronbach’s alpha)	Items	Item loadings	CR	AVE
Biospheric (*α* = 0.909)	1. Respecting the earth 2. Unity with nature 3. Protecting the environment 4. Preventing pollution	0.907 0.785 0.922 0.813	0.918	0.737
Altruistic (*α* = 0.846)	1. I have given directions to a stranger 2. I have given money or donated goods to a charity 3. I have given money to a stranger who needed it 4. I have pointed out a clerk’s error in under charging me for an item 5. I have let a neighbor whom I did not know too well borrow an item of some value to me 6. I have offered my seat on a bus or train to a stranger who was standing	0.666 0.748 0.796 0.661 0.681 0.713	0.860	0.508
Collectivistic (*α* = 0.924)	1. I like to work hard for the accomplishment of goals of my group 2. I like to help others in a time of need 3. I like to maintain good relationships with others 4. To do well in life, the help of friends is crucial 5. One of the pleasures in life is to be interdependently related to others	0.845 0.884 0.902 0.832 0.770	0.927	0.719
GPA (*α* = 0.947)	For me, purchasing a green car is – 1. Good 2. Desirable 3. Pleasant 4. Wise	0.867 0.851 0.908 0.917	0.936	0.785
Subjective norm (*α* = 0.961)	1. Most people who are important to me think I should purchase a green car 2. Most people who are important to me would want me to purchase a green car 3. People whose opinions I value would prefer that I purchase a green car	0.960 0.965 0.913	0.963	0.895
PBC (*α* = 0.815)	1. Whether or not I purchase a green car is entirely up to me 2. I am confident that if I want, I can purchase a green car 3. I have resources, time and opportunities to purchase a green car	0.740 0.853 0.772	0.832	0.624
GPI (*α* = 0.932)	1. I am willing to purchase a green car in future 2. I will make an effort to purchase a green car in future 3. I plan to purchase a green car	0.925 0.922 0.875	0.933	0.824
Function value (*α* = 0.948)	1. Green car is cozy and comfortable 2. Green car is technically innovative 3. Green car is environmentally friendly 4. Green car gets good mileage 5. Green car preserved some traditional facets 6. The overall green car experience is value for money 7. Green car was accessible 8. The green car staff were friendly and courteous 9. The green car staff were able to converse well 10. The green car program is an economical car-using package	0.699 0.674 0.694 0.833 0.858 0.779 0.866 0.756 0.858 0.846	0.943	0.623
Emotional value (*α* = 0.885)	1. There is a feeling of individuality about take/drive green cars 2. Take/drive green car is exciting 3. Take/drive green cars make me funny and enjoyable 4. Green car was something new and different 5. My experience take/drive green car was something special and relaxing	0.928 0.957 0.948 0.533 0.585	0.902	0.660
Social value (*α* = 0.815)	1. I will feel proud of my green car 2. Purchasing green car was a smart choice 3. Purchasing green car helps me to feel acceptable to others 4. Purchasing green car enables me to impress others	0.777 0.881 0.698 0.501	0.812	0.529

**Table 3:** Discriminant validity of measurement model (HTMT test).

S. No.	Items	1	2	3	4	5	6	7	8	9	10
1.	BV	***1.000***									
2.	AV	0.401	***1.000***								
3.	CV	0.612	0.694	***1.000***							
4.	GPA	0.602	0.436	0.546	***1.000***						
5.	PBC	0.414	0.490	0.446	0.625	***1.000***					
6.	SN	0.243	0.486	0.376	0.638	0.763	***1.000***				
7.	GPI	0.472	0.539	0.566	0.806	0.732	0.740	***1.000***			
8.	FV	0.482	0.638	0.663	0.610	0.576	0.601	0.665	***1.000***		
9.	EV	0.543	0.601	0.678	0.771	0.641	0.673	0.842	0.779	***1.000***	
10.	SV	0.106	0.118	0.126	0.209	0.176	0.222	0.229	0.229	0.177	***1.000***

*EV, Emotion value; BV, Biospheric value; AV, Altruistic value; CV, Collectivistic value; GPA, Green purchase attitude; PBC, Perceived behavioral control; SN, Subjective norm; GPI, Green purchase intention; FV, Function value; SV, Social value*.

The model fit indices showed that the measurement model contained an adequate fit to the data: *X*^2^ = 3070.803, DF = 979, *p* < 0.001, CMIN/DF = 3.137 (below guidelines of 2–5), RMR = 0.068, CFI = 0.915, IFI = 0.915, TLI = 0.906, PGFI = 0.697, PNFI = 0.797, PCFI = 0.829, RMSEA = 0.063, PCLPSE <0.001.

#### Structural Model Estimation

**Figure 2:**
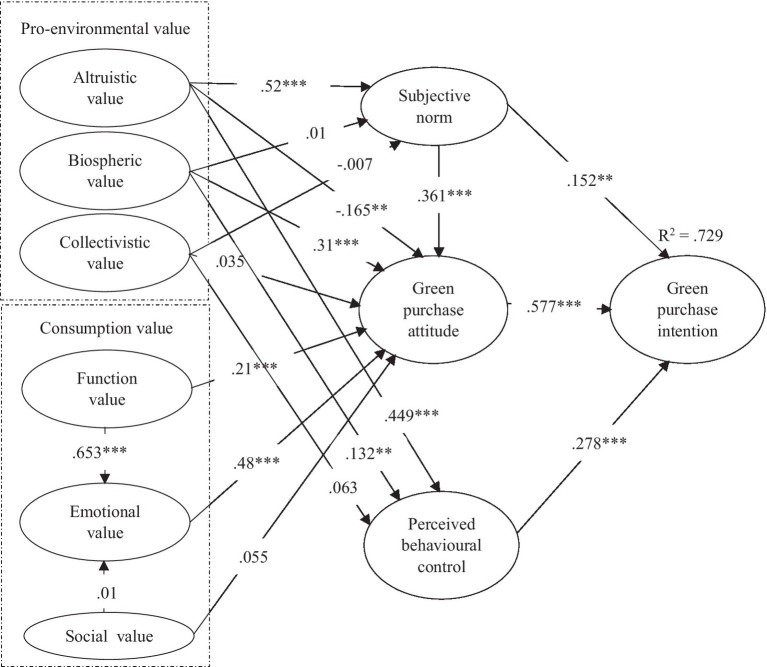
Structural model results. ^**^p < 0.01, ^***^p < 0.001, and Critical ratio > 1.96.

Structural equation modeling (SEM) was performed to test the hypotheses of this study. Indeed, for mediation test, the bias-corrected percentile method (BC) was considered as it is still the most accurate method implemented in the software package (Carpenter and Bithell, 2000). Thus, the results of mediational relationships will be observed based on direct and indirect values from two-tailed significance under bootstrap confidence, and the test turned out to be significant at the *p* < 0.05 level. In the model fit summary, *X*^2^ = 3241.270, DF = 990, *p* < 0.001, CMIN/DF = 3.274, CFI = 0.909, IFI = 0.909, TLI = 0.900, PGFI = 0.703, PNFI = 0.800, PCFI = 0.832, RMSEA = 0.065, PCLPSE <0.001. Details about the structural model evaluation results are illustrated in Figure 2 and Table 4, accordingly.

**Table 4:** Results of the structural model.

Hypothesized paths	*β*	C.R.	Sig.	Decision
H1: Altruistic value → GPA	−0.165	−2.670	0.008	Not supported
H2: Altruistic value → SN	0.520	7.612	^***^^c^	Supported
H3: Altruistic value → PBC	0.449	5.913	^***^^c^	Supported
H4: Biospheric value → GPA	0.310	8.177	^***^^c^	Supported
H5: Biospheric value → SN	0.010	0.221	0.825	Not supported
H6: Biospheric value → PBC	0.132	2.630	0.009	Supported
H7: Collectivistic value → GPA	0.035	0.653	0.513	Not supported
H8: Collectivistic value → SN	−0.007	−0.109	0.913	Not supported
H9: Collectivistic value → PBC	0.063	0.869	0.385	Not supported
H10: Function value → GPA	0.210	3.642	^***^^c^	Supported
H11: Emotional value → GPA	0.480	10.743	^***^^c^	Supported
H12: Function value/emotional value/GPA			0.019^a^ 0.000^b^	Supported
H13: Social value → GPA	0.055	1.669	0.095	Not supported
H14: Social value/emotional value/GPA			0.059^a^ 0.715^b^	Not supported
H15: Green purchase attitude → GPI	0.577	14.639	^***^^c^	Supported
H16: Subjective norm → GPI	0.152	2.760	0.006	Supported
H17: Subjective norm/GPA/GPI			0.108^a^ 0.001^b^	Supported
H18: Perceived behavioral control → GPI	0.278	4.474	^***^^c^	Supported

a *Denotes standardized direct effect with bootstrapping method (two-tailed significance)*.

b *Denotes standardized indirect effect with bootstrapping method (two-tailed significance)*.

c Denotes *p* < 0.001.

## Conclusion and Discussion

This study focused on the both the pro-environmental value and the consumption value on the belief-related aspects (i.e., SN, PBC, and GPA) and behavioral aspect (i.e., GPI) toward green car purchasing behavior. An integrated value–attitude–behavior model and TPB model that incorporated altruistic value, biospheric value, collectivistic value, function value, emotional value and social value, SN, GPA, PBC and GPI was developed and empirically tested.

According to Wang and Wong (2021), a path coefficient of below 0.1 means a small effect; a path coefficient of about 0.3 indicates moderate effect; and a path coefficient of 0.5 and above shows a large effect. Previous studies showed that there is a positive relationship between altruistic value and GPA (Birch et al., 2018; Wang et al., 2020a). However, the results indicated that altruistic value moderately and negatively influenced GPA (*β* = −0.165, *p* < 0.01). This means that the young Chinese generation’s GPA toward purchasing a green car was generated without considering the benefits of the purchase to others. Individuals may look for self-interest in advance of desire to benefit to others *via* purchasing green cars. According to Wang et al. (2020a), studies on the effect of altruistic value in green marketing are scarce when compared to conventional studies. This study may conclude that young consumers GPA will be negatively connected with their altruistic value exceed other values when they purchase high-interest products. Thus, H1 was not supported. However, altruistic value positively influenced SN (*β* = 0.52, *p* < 0.001) and PBC (*β* = 0.449, p < 0.001) respectively. It can be said that the Chinese young generation is generally more concerned about their significant referents’ opinions and their perceived ability to purchase green cars which is highly influenced by their concern about other (environmental) benefit. This result is supported by Obrenovic et al. (2020) and Teng et al. (2015) who argued that altruistic value has statistical impact on GPA and PBC, respectively. Hence, H2 and H3 were supported.

Based on previous studies, the biospheric value has been proven to have a significant positive influence on GPA in green marketing (Rahman and Reynolds, 2016; Wang et al., 2021b), and in some circumstance, individuals concern about environment played the most important role in determining one’s green purchase propositions (Jaiswal and Kant, 2018; Wang, 2020). The results of this study show that there is a positive significant relationship between biospheric value and GPA (*β* = 0.31, *p* < 0.001). This means that the young generation who is more concerned about the environment will possess a higher positive attitude to purchase green cars in future. Young consumers concern about physical environment dominated in their GPA compared to other type of environmental values toward purchasing green cars, and thus, H4 was supported. The result of the study also suggests that the biospheric value positively influences PBC (*β* = 0.132, *p* < 0.01). This result also stands in line with some studies showing that the biospheric value positively influences PBC (Rahman and Reynolds, 2016, 2019). It can, therefore, be claimed here that individuals’ concern about ecosystem will lead to a stronger perception of their abilities to overcome barriers to purchase green cars. This shows that they may spend more time, information, and resources on looking for green cars as they would like to preserve natural resources. Thus, H6 was supported. However, the results of this study also indicate that the biospheric value non-significantly influence SN. This result stands in contrast to some studies showing that the biospheric value positively influence on SN (Bamberg, 2003; Paul et al., 2016). This means that the Chinese young generation are not particularly concerned about their significant referents’ opinions’ influence on their own biospheric value. It can be said that individuals’ environmental concern and awareness were shaped by their environmental beliefs, and this not easily can be influenced from their significant others’ suggestions or word of mouth. Hence, H5 was not supported.

Furthermore, it is a surprise that collectivistic value has no role in this study as it did not influence GPA, SN, or PBC. These results differ from previous studies (Kumar and Sreen, 2020; Wang et al., 2020a; Wang and Wong, 2021) which showed that collectivistic value should play a significant role in determining pro-environmental behaviors, specifically in eastern countries (e.g., China). One reason may due to the fact that the young generation is becoming more individualistic when compared to their older counterparts (Wiratno et al., 2020). Another reason may be that product-related attributes (e.g., function value and emotional value) play a more significant role in determining their green car purchasing behavior over collectivistic value due to the results showed that consumption values had more influence compared to pro-environmental values. Thus, H7, H8, and H9 were not supported.

In prior studies, function value has been proven to be an important predictor that led to positive attitude and intention in green marketing (Sierzchula et al., 2014; Bjerkan et al., 2016). This study also revealed a moderately significant positive relationship between function value and GPA (*β* = 0.21, *p* < 0.001). The Chinese young generation concerns about green cars’ functional, utilitarian, and physical performance attributes greatly influenced their attitudes toward purchase green cars. Hence, H10 was supported. This study also demonstrated a moderate significantly positive correlation between emotional value and GPA. These results are consistent with those reported in previous studies that suggest emotional value is an important predictor for consumers’ GPA and behavior (Rasoolimanesh et al., 2020; Joshi et al., 2021). It means that individuals’ experience and feeling about owning green cars (e.g., hedonic orientation, exciting, and novelty) compared to conventional cars can lead to positive responses, thus, significantly influencing their GPA. Hence, H11 was supported. According to Hur et al. (2013), consumers may feel connected and belonging to an environmentally conscious group *via* purchasing hybrid cars, and thus, benefit others. Nevertheless, this study did not show any significant relationship between social value and GPA toward green car purchase intention. It can be explained that the Chinese young generation did not feel that they belong to any environmentally conscious groups *via* purchasing green cars, thus, social value is not a significant predictor for their green car purchasing attitude. This result was also somewhat reflective of the earlier finding that collectivistic value did not influence the Chinese young generation’s green car purchase attitude. The social pressure on environmental protection and group goal for preserving natural resources *via* purchasing green cars cannot influence young Chinese consumers to make decisions for high-interest products (i.e., green cars). Hence, H13 was rejected.

Babin et al. (2004) mentioned that consumers’ functional value attributes positive effects on hedonic shopping values and service quality is positively correlated with emotional value. The results of this study show that the direct link between function value and GPA (*p* < 0.05), and the indirect relationship between function value and GPA through emotional value (*p* < 0.001) was found to be statistically significant. This result suggests that emotional value plays a partially mediating role in the relationship between functional value and GPA. Therefore, H12 was supported. Furthermore, Nkaabu et al. (2017) argued that social value positively influenced consumers’ evaluation of emotional value generated toward purchase intention. However, findings from this study indicated that the direct relationship between social value and GPA (*p* > 0.05) and the indirect relationship between social value and GPA *via* emotional value (*p* > 0.05) were found to be statistically insignificant. This is reflective of earlier results which revealed that there is an insignificant relationship between social value and GPA. Hence, emotional value has no role in mediating the relationship between social value and GPA, and H14 was rejected.

The results showed that GPA has a major influence on intention (*β* = 0.577, *p* < 0.001). This result is consistent with those reported in previous studies that GPA is the most important variable that influenced intention and behavior (Jaiswal and Kant, 2018; Wang et al., 2019). This means that the Chinese young generation who have more positive attitude toward green cars will possibly select a green car as their transportation tool in future. Therefore, H15 was supported. In prior studies, SN has been proven to be an unstable predictor of GPI even though certain studies showed SN positively influenced GPI (Bahl and Kumar, 2019; Liu et al., 2020), a number of other studies demonstrated that SN has a non-significant relationship with GPI (Patharia et al., 2020; Wang and Wong, 2021). The current study’s results indicated that SN has a moderate significant (*β* = 0.152, *p* < 0.01) influence on GPI. This shows that when Chinese young generation receive important opinions about green cars from significant others, it can positively influence their green car purchasing intention. Hence, H16 was supported. In some circumstances, PBC may play the most important role in determining GPI in TPB (Zhou et al., 2013). In addition, certain studies showed that GPI is positively influenced by consumers’ PBC (Ateş, 2021; Lin et al., 2021). In other words, the more confident the consumers have in overcoming barriers (e.g., resources, money, and time) in their quest to purchase green cars, the more likely they will engage in such purchasing behavior. This study confirms that there is a moderate significant relationship between PBC and GPI (*β* = 0.278, *p* < 0.001). Hence, H18 was supported. Furthermore, Wang et al. (2021b) indicated that the significant other’s views on the GPB performance can influence one’s attitude, and subsequently on their intention among eastern cultures. Researchers should consider the significant causative path from SN to attitude in eastern nations (Wang and Wong, 2021). This study subsequently confirms the direct relationship between SN and GPI was statistically insignificant (*p* > 0.05), while the indirect link between SN and GPI *via* GPA was found to be significant (*p* < 0.01); which denotes GPA fully mediates the relationship between SN and GPI. Thus, H17 was supported.

### Theoretical Contributions

First, this study is among the first that empirically tested and validated the significant relationships of altruistic value, biospheric value, collectivistic value, functional value, emotional value, social value, SN, GPA, PBC, and intention toward green car purchase behavior based on a merged VAB and TPB models. TPB model assumes that self-interests, including weighted expected cost and benefits of alternatives (e.g., time, money, opportunities, and social approval) motivates individuals (Lindenberg and Steg, 2007). Thus, TPB had more explanatory power in high behavioral cost or strong constraints situations when compared to value orientation theories (e.g., value-belief-norm theory; Wang et al., 2021b). But at the same time, the nature of TPB is its focus on rational reasoning and it lacks consideration on personal decision criteria, such as subconscious, feelings, and private standards (Ulker-Demirel and Ciftci, 2020). In fact, the nature of the relationship between values, beliefs, attitude and behavior is complex (Wang and Wong, 2021). Tamar et al. (2020) showed value is considered as a stable trans-situational goal which varies in degree of importance and serve as a guiding principle in one’s life, and as one of the critical antecedent for GPB (Wang et al., 2021b). However, compared to TPB model, such theories (e.g., value-belief-norm theory, VAB) were found to have less explanatory power in explaining high-cost GPB (Wang et al., 2021b). By investigating the influence of various aspects of pro-environmental values (i.e., collectivistic value, altruistic value, and biospheric value), consumption values (i.e., functional value, emotional value, and social value) and components of TPB (i.e., attitude, SN, and PBC) on GPI toward green cars purchasing, this study offers a more comprehensive perspective on the green cars purchasing behavior among young generations.

Second, although consumption values have been adopted in various studies in the literature (Jan et al., 2019; Zaidi et al., 2019; Caber et al., 2020), the link between consumption values and behavior does not seem to explain behavior comprehensively in green marketing (Jacobs et al., 2018). This study enriches the understanding of how function value, emotional value, and social value influence GPA, and ultimately the effect of GPI toward green car purchase behavior in China. Previous studies indicated that in some circumstances, consumers’ emotional value can be influenced by their functional value or social value (Ladhari et al., 2017; Rasoolimanesh et al., 2020). The results from this study confirmed that emotional value plays a full mediation role between function value and GPA. This lends support to the argument that function value has a significant effect on attitude *via* emotional value toward certain purchasing behavior (Ladhari et al., 2017; Rasoolimanesh et al., 2020). Nevertheless, the results of this study did not confirm that emotional value mediates the relationship between social value and attitude.

Third, this study provides an exhaustive understanding of the influence of pro-environmental values on attitude, SN, and PBC. Although the VBN model successfully applied altruistic value, biospheric value, and egoistic value in predicting GPB in previous studies (Wang et al., 2020a, 2021d), there were certain gaps that need to be addressed. First, studies on altruistic value are scarce compared to conventional studies (Wang et al., 2020a); second, most previous studies related to GPB have not distinguished biospheric value from altruistic value (Rahman and Reynolds, 2016); and third, egoism does not seem to be a suitable predictor for consumers in eastern cultures with collectivistic values (Wang et al., 2020a). The results from the current study indicated that altruistic value positively influenced SN and PBC, but negatively influenced GPA; biospheric value positively influenced GPA and PBC; meanwhile, collectivistic value had no influence on GPA, SN, and PBC toward green cars purchasing. The obtained results offer an alternative perspective on the consumers’ pro-environmental values and green cars’ purchase intention and offers valuable insights on the influence of altruistic value, biospheric value, and collectivistic value on GPA in an eastern country.

Last, the TPB model is prevalently applied in green marketing studies. However, there is a lack of understanding of Chinese consumers’ GPB in studies using western samples (Ulker-Demirel and Ciftci, 2020). The results of this study showed that the components of TPB (SN, GPA, PBC) undoubtedly played a significant role in determining GPB toward green car purchasing behavior. In addition, certain studies demonstrated that the possible mediation effect of SN in the TPB model cannot be ignored (Bashir et al., 2019; Wang and Wong, 2021). This study confirmed that GPA played a mediation role between SN and GPI. More importantly, the obtained results confirmed the existence of certain relationships between individuals’ pro-environmental values, consumption values, SN, GPA, PBC, and GPI toward green car purchasing behavior. This study offers an alternative perspective on the individual’s values and beliefs toward green car purchasing intention among the young generation.

### Practical Implications

The results of this study indicated that altruistic value, collectivistic value, and social value had negative or no influence on consumers’ GPA. In contrast, biospheric value, functional value, and emotional value had positive influence on their GPA. Young generations are becoming more individualistic and independent in marking decisions to purchase some novel products and services. When they purchase high-cost products (i.e., green cars), they are not unduly concerned about benefits to others *via* their green car purchase; they do not feel that they are connected to others who have the same goal of protecting the environment by purchasing or using green cars.

Meanwhile, they are more concerned about the green cars’ applicable attributes (e.g., innovation, cost-saving, good mileage, and comfortable), and on the feelings of using/driving green cars (e.g., exciting, enjoyable, and uniqueness). Thus, the green car industry should pay more attention on its products; for example, they can promote key characteristics of their green cars: how they can save money compared to traditional cars, and they can disseminate information on the latest innovations they have adopted in green car technology (e.g., energy recovery, noiseless, short-charging time, non-maintenance, affordable, and cozy). At the same time, they can educate the consumers on how they can protect the environment *via* purchasing green cars (e.g., non-pollution and non-greenhouse gas emissions). Finally, highlighting the emotions of driving a green car (pleasure, excitement, thrill, trendy) is vital for green car manufacturers, as emotional value has a significant impact on consumers’ GPA, followed by biospheric value and functional value.

Second, this study shows that collectivistic value had no influence on consumers’ SN and PBC in a highly collectivistic society (i.e., China). It means that consumers’ intention to purchase green cars is not influenced by group goals to protect environment. In addition, biospheric value had no influence on SN. This means that young Chinese consumers are becoming more individualistic and less concerned about the opinions of others on environmental consciousness. Meanwhile, altruistic value positively influenced one’s SN and PBC, and biospheric value positively influenced PBC. This means that consumers are more concerned about how purchasing green cars can lead to positive feedback from their significant others. Thus, by highlighting the green functional attributes of green cars and how they can benefit the consumers’ referent groups *via* purchasing green cars can lead to greater environmental awareness among the youth market. Evoking consumers’ environmental concerns and benevolence to others is another strategy for the green car industry to promote their products.

Third, as GPA, PBC and SN were found to significantly influence GPI, it is important to recognize that positive attitudes, significant others’ opinions, as well as a high level of confidence to overcome obstacles were important influencing factor for young potential consumers to purchase green cars. Green car manufacturers should convey the message that green cars purchase can help protect the environment for themselves and others, and at the same time, highlighting the ease of owning green cars (such as saving money in the long run due to reduced fuel costs and ease of charging); thus, eliminating some of the perceived barriers to purchase green cars. The young generation have never lived without the internet (Kusumawati et al., 2019), therefore it is imperative that both online (e.g., web-based advertisings) and traditional media are used in promotional campaigns for green car manufacturers to effectively reach this target segment.

Last but not least, as GPB awareness is still at its infancy stage in China (Wang and Wong, 2021), pro-environmental education needs to be provided by the green car industry and should be targeted to the young generation as they are more receptive and concerned about environmental issues. Information that should be disseminated to these potential consumers can include information on how the adoption of green vehicles in the transportation section can lead to a significant reduction in the emission of greenhouse gases and CO_2_ emissions, which subsequently, will lead to an increase in the young generation’s biospheric value and environmental awareness.

### Limitation and Future Recommendations

First, as this is a cross-sectional study, no definite conclusions can be drawn on the causality of the relationships in this research. Second, the sample for this study was selected from young university students, which is not representative of the entire Chinese population and therefore the results are not generalizable. Indeed, certain studies demonstrated that specific student samples could be detected showing a variety of environmental attitude and dispositions (Lambrechts et al., 2018; Caniëls et al., 2021), and replicating student results on non-students could be a huge challenge for researchers in any disciplines (Henry, 2008). Future research should examine the effect of pro-environmental value and consumption value from divergent populations on GPB. Lastly, although intention may be robust in certain behavioral studies, an individual’s actual behavior is not always reflective of one’s stated behavioral intention (Wang et al., 2021c). Hence, the influence of behavioral intention on Chinese young consumers’ actual green cars purchasing/GPB should be further investigated in future research.

## Data Availability Statement

The raw data supporting the conclusions of this article will be made available by the authors, without undue reservation.

## Ethics Statement

The studies involving human participants were reviewed and approved by Business School, Xuzhou University of Technology. Written informed consent from the participants’ legal guardian/next of kin was not required to participate in this study in accordance with the national legislation and the institutional requirements. Written informed consent was not obtained from the individual(s), nor the minor(s)’ legal guardian/next of kin, for the publication of any potentially identifiable images or data included in this article.

## Author Contributions

LW contributed to the design of the work, data collection, data analysis and interpretation, drafting the article, and final approval of the version to be published. PW contributed to the data interpretation, drafting the article, critical revision of the article, and final approval of the version to be published. QZ contributed to the data collection and data analysis and interpretation. All authors contributed to the article and approved the submitted version.

## Conflict of Interest

The authors declare that the research was conducted in the absence of any commercial or financial relationships that could be construed as a potential conflict of interest.

## Publisher’s Note

All claims expressed in this article are solely those of the authors and do not necessarily represent those of their affiliated organizations, or those of the publisher, the editors and the reviewers. Any product that may be evaluated in this article, or claim that may be made by its manufacturer, is not guaranteed or endorsed by the publisher.
